# Importância da Técnica Anestésica e Analgesia no Implante de Desfibrilador Subcutâneo e Endovascular: Um Aspecto Geralmente Ignorado

**DOI:** 10.36660/abc.20210184

**Published:** 2021-06-08

**Authors:** Jorge Elias

**Affiliations:** 1 Vitória Apart Hospital SerraES Brasil Vitória Apart Hospital - Serviço de Eletrofisiologia, Serra , ES – Brasil

**Keywords:** Taquiarritmia Ventricular, Cardioversor-Desfibrilador implantável/tendências, CDI subcutâneo/tendências, Qualidade de vida, Sedação Consciente, Anestesia, Analgesia

Originalmente introduzido por Mirowski há 50 anos, o cardioversor-desfibrilador implantável (CDI) tornou-se um dos pilares na prevenção de MSC relacionada a taquiarritmias ventriculares. ^1^

Entretanto, além da capacidade de salvar vidas, os cateteres transvenosos têm seus próprios riscos.

As inovações tecnológicas e a observação dessas complicações e limitações levaram ao surgimento do CDI subcutâneo (CDI-S). ^1^

Essa técnica está evoluindo rapidamente para se tornar uma alternativa segura e eficiente para o CDI-TV, deixando o coração e a vasculatura intocados, e reduzindo complicações relacionadas a cateteres. ^1 - 5^

Novas intervenções e dispositivos necessariamente demandam uma análise constante da técnica anestésica apropriada a ser usada. Apenas recentemente, os primeiros estudos que lidam com o implante de CDI-S surgiram e analisaram a questão da segurança perioperatória e analgesia pós-operatória e seu impacto no paciente. Entretanto, como os autores observaram, há uma carência de estudos formatados que comparem esses dois tipos de CDI.

Nessa edição, Auquilla-Clavijo et al. ^6^ lidam com essas duas questões fundamentais: comparar a QDV e a percepção da dor e do desconforto resultante da técnica cirúrgica (e, principalmente, o protocolo anestésico usado), levando em consideração o tipo de dispositivo implantado no paciente (CDI-TV vs. CDI-S). ^6^

Como relatado, os autores usaram a técnica anestésica chamada de sedação e analgesia administrada por médicos não anestesiologistas ( *non-anesthesiologist-administered sedation and analgesia* - NASA). ^6^

Embora ela seja segura e perfeitamente viável, não se pode considerar que os resultados sejam reproduzidos em serviços que contam com uma equipe dedicada de anestesiologistas. Ou seja, os resultados são válidos para situações semelhantes, mas não se exclui a possibilidade de se obter um resultado mais favorável para a pesquisa sobre QDV durante a sedação realizada pela equipe de anestesia.

Na verdade, há hesitação em relação ao uso de anestesia local com sedação consciente para procedimentos implante de terapia de ressincronização cardíaca com desfibrilador (TRC-d) ou CDI-S. ^7^ Essa questão é plausível se forem consideradas as diferenças significativas entre a gestão perioperatória do CDI-S e do CDI-TV. ^1^ (Ref 2) O CDI-S requer uma dissecção de tecido mais extensa, tunelização de cateter, e, apesar dos resultados recentes com o escore PRAETORIAN, o teste de desfibrilação (TD) continua sendo a rotina. ^4 , 6^

Ainda não se sabe qual é o melhor anestésico para o implante de CDI-S e TD, já que há uma escassez de dados randomizados. Entretanto, uma análise da literatura demonstra a eficácia e a segurança do implante de CDI-S utilizando-se várias modalidades: anestesia geral (AG); cuidado anestésico monitorado (MAC), um serviço administrado por um médico ou enfermeiro anestesista; anestesia regional e local suplementadas com técnicas de sedação/analgesia. ^8^ Provavelmente, um procedimento bem-sucedido pode ser realizado com uma variedade de modalidades de anestesia que devem levar em consideração os aspectos clínicos e as comorbidades dos pacientes, bem como a experiência e a preferência da equipe médica. ^8^

A AG pode ser utilizada, mas não é necessária para implantes de CDI-S, e deve-se, sempre que possível, optar pelo MAC. Se a equipe, como neste artigo, optar pela abordagem NASA durante o implante de CDI, sugere-se que seja realizada uma fase de curva de aprendizado (os primeiros 5-10 implantes), e a realização de um programa de treinamento apropriado. O treinamento deve incluir políticas e procedimentos para orientar a administração de sedação, o monitoramento de pacientes, e a gestão de vias aéreas. ^8^

A recente incorporação de técnicas de bloqueio de nervo peitoral, chamadas PECS I e II, guiadas por ultrassom, proporcionam a anestesia do músculo transverso do tórax, e de bloqueios do músculo serrátil anterior, cobrindo tanto a região torácica anterior (inclusive os músculos peitorais maior e menor), bem como anestesia o nervo intercostobraquial, os nervos intercostais de três a seis, e o nervo torácico longo. ^1 , 8 - 10^ As vantagens dos bloqueios de nervo peitoral são sua rapidez e facilidade de anestesia. Entretanto, mesmo com o bloqueio regional adequado, os pacientes que passam por CDI-S podem precisar também de AG ou MAC, porém, possivelmente, em um grau menor.

Outra questão relevante está relacionada ao protocolo (medicamentos e as respectivas doses) utilizado para a sedação consciente. A benzodiazepina (nesse caso, midazolam) está associada a delírios, especialmente em pacientes idosos; e o propofol em bomba de infusão proporciona um despertar melhor, menos náusea e vômito pós-operatório, e menos tempo de recuperação pós-anestésica. ^7 - 9^

Sabe-se que a mortalidade de recipientes de CDI é significativamente prevista por sua qualidade de vida (QDV). Uma meta-análise recente demonstrou que intervenções psicoeducativas melhoram o componente físico, mas não o componente mental da QDV em pacientes com CDI. Esse ponto também é relevante em relação aos resultados a serem obtidos com a percepção da intervenção (independentemente do tipo de dispositivo implantado). ^11^

O que o estudo também deixa claro é que a condição mental (psicológica, tipo de personalidade) do paciente interfere diretamente nos resultados obtidos nos questionários comumente utilizados.

Essa observação leva a outro aspecto a ser considerado: cerca de 20% dos pacientes submetidos a implante de CDI apresentam sintomas de depressão. O aspecto psicológico é fundamental, entretanto, uma vez que os sintomas de depressão afetam não apenas a qualidade de vida dos pacientes, como também aumentam seu risco de morte prematura apesar do tratamento de ponta com o CDI. ^12^

Estudos mostram que a pesquisa sobre a personalidade tipo D é possivelmente fundamental, já que é um preditor independente da depressão pós-implante e pode comprometer os resultados de estudos que não tentam investigar essa variável. Outro aspecto é que a predominância de homens nesse estudo pode comprometer o nível de atitude positiva em relação à dependência da tecnologia, dificultando a generalizabilidade.

No presente estudo, foi utilizado o questionário SF-12 (Formulário curto de 12 itens para pesquisa de saúde). Sabe-se que o SF-12, bem como o SF-36, são os indicadores de QDV mais amplamente utilizados em estudos da população com CDI internacionalmente. ^13^ Entretanto, o SF-12 pode não detectar resultados de QDV específicos do CDI, especialmente o bem-estar mental, e esse é um aspecto limitador desse estudo. ^11^

Por exemplo, o estudo EFFORTLESS S-ICD Registry, além de usar o SF-12 teve o cuidado de evitar vieses que pudessem ser atribuídos ao tipo de personalidade do paciente. Para isso, foi utilizado o DS 14. Os autores tomaram esse cuidado, como indicado acima, por terem o conhecimento de que a personalidade tipo D é um fator de vulnerabilidade para QDV pior, arritmias potencialmente fatais, e mortalidade prematura em pacientes com CDI. ^14^

Outra possibilidade seria o autor utilizar a versão SF-12v2, que também inclui 2 sínteses estatísticas, incluindo um escore físico ( *Physical Component Summary* ou PCS) e um escore mental ( *Mental Component Summary* ou MCS). ^11 , 15^

Por último, devido ao importante viés resultante de pacientes operados novamente após complicações prévias com um dispositivo CDI-TV, considerou-se que a análise comparativa de QDV entre grupos não pode ser avaliada.

Espera-se que o aumento contínuo de CDI-S e estudos prospectivos consiga reproduzir essas observações unicêntricas e determinar a melhor forma de proporcionar uma técnica anestésica de implante segura, eficiente e confortável, concentrando-se sempre no impacto na QDV dos pacientes.
