# Supplementary material for: Importância da Técnica Anestésica e Analgesia no Implante de Desfibrilador Subcutâneo e Endovascular: Um Aspecto Geralmente Ignorado
Source: Arq Bras Cardiol. 2021 Jun 8;116(6):1150–2. [Article in Portuguese] doi: 10.36660/abc.20210184 (PMC8288522; doi:10.36660/abc.20210184)
Supplement: Translation

# Translation: Importance of the Anesthetic Technique and Analgesia in the Implantation of Subcutaneous and Endovascular Defibrillator: An Aspect Often Ignored

**Authors:** Jorge Elias

**Affiliations:** 1 Brasil Vitória Apart Hospital - Serviço de Eletrofisiologia, Serra, ES – Brasil

**Keywords:** Tachycardia, Ventricular, Cardioversor–Defibrillator Implantable/trends, CDI Subcutaneous/trends, Quality of Life, Conscious Sedation, Anesthesy, Analgesia

Originally introduced by Mirowski 50 years ago, the implantable cardioverter-defibrillator (ICD) has become a cornerstone to preventing SCD related to ventricular tachyarrhythmias.^1^

However, besides their lifesaving capacities, the transvenous leads carry their own risk.

The technological improvement and the observation of these complications and limitations led to the appearance of the subcutaneous ICD (S-ICD).^1^

This technique is rapidly evolving to become a safe and effective alternative for the TV-ICD, leaving the heart and vasculature untouched, with reduced lead-related complications.^1 - 5^

New interventions and devices necessarily demand a constant analysis of the appropriate anesthetic technique to be used. Only recently, the first studies that deal with the implantation of S-ICD have appeared and analyzed the issue of perioperative safety and postoperative analgesia, as well as its impact on the patient. However, as the authors have pointed out, there is a lack of formatted studies to compare these two types of ICD.

In this edition Auquilla-Clavijo et al.,^6^ address these two fundamental questions: comparing QOL and the perception of pain and discomfort resulting from the surgical technique (and, more importantly, the anesthetic protocol used), taking into account the type of device implanted in the patient (TV-ICD x S -ICD).^6^

As reported, the authors used the anesthesiological technic, called non-anesthesiologist-administered sedation and analgesia (NASA).^6^

Although safe and perfectly feasible, it is not possible to consider that the current results are reproduced in services that have a dedicated team of anesthetists. That is, the results are valid for similar situations but do not rule out the possibility of obtaining a more favorable result in QOL research during sedation conducted by the anesthesia team.

In fact, there is some hesitation regarding the use of local anesthesia with conscious sedation for cardiac resynchronization defibrillator therapy (CRT-d) or S-ICD implantation procedures.^7^ This question is plausible if we consider the significant differences between the perioperative management of the S-ICD and the TV-ICD^1^ (Ref 2). The S-ICD requires more extensive tissue dissection and tunneling of a lead, and despite recent results with the PRAETORIAN SCORE, defibrillation testing (DT) remains routine.^4 , 6^

The best anesthesia for S-ICD implantation and DT is unknown, as a paucity of randomized data exists. However, a review of the literature demonstrates efficacy and safety for S-ICD implantation using several modalities: general anesthesia (GA); Monitored Anesthesia Care (MAC), a service provided by an anesthesiologist or certified registered nurse anesthetist; and regional anesthesia and local anesthesia supplemented with sedation/analgesia techniques.^8^ A successful procedure can most likely be accomplished with a variety of anesthesia modalities that must take into account clinical aspects and comorbidities of patients, as well as the experience and preference of the medical team.^8^

GA can be used, but it is not required for S-ICD implants, and professionals should, whenever possible, choose MAC. If the team, as in this article, opts for the NASA approach during the ICD implant, both a learning curve phase (initial 5-10 implants) and the completion of an appropriate training program are suggested. This training should include policies and procedures to guide the administration of sedation, patient monitoring, and airway management.^8^

The recent incorporation of truncal plane nerve block techniques, called PECS I&II, guided by ultrasound, provides anesthesia to the transversus thoracic muscle plane, and the serratus anterior plane blocks, covering both the anterior thoracic region (including the pectoralis major and minor muscle) as well as anesthetizes the intercostobrachial nerve, intercostal nerves three through six, and the long thoracic nerve.^1 , 8 - 10^ The advantages of truncal plane blocks are that they are quick and easy for anesthesia. However, even with adequate regional block, patients undergoing S-ICD may still require GA or MAC but perhaps to a lesser extent.

Another relevant issue concerns the protocol (drugs and respective doses) used for conscious sedation. Benzodiazepine (in this case midazolam) is associated with delirium, particularly in elderly patients; and propofol in infusion pumps produces better arousal, less postoperative nausea and vomiting, and a shorter post-anesthetic recovery time.^7 - 9^

We know that ICD recipients’ mortality is significantly predicted by their quality of life (QOL). A recent meta-analysis has shown that psycho-educational interventions improve the physical component but not the mental component of QOL in patients with ICD. This point is also relevant concerning the results to be obtained with the perception of the intervention (regardless of the type of implanted device).^11^

What this study also makes clear is that the patient’s mental (psychological, personality type) situation directly interferes in the results obtained in the commonly used questionnaires.

This observation leads us to another aspect that we would like to consider: about 20% of the patients submitted to the ICD implant show symptoms of depression. The psychological aspect is fundamental, however, given that depressive symptoms not only affect patients’ quality of life, but also increase their risk of premature death despite state-of-the-art treatment with the ICD.^12^

Studies show that D-type personality research is possibly essential, since it is an independent predictor of post-implant depression and may compromise the results of studies that do not attempt to investigate this variable. Another aspect is that the predominance of males in this study may compromise the degree of positive attitudes toward technology dependency, thus making generalizability difficult.

In the current study, the SF-12 questionnaire (12-Item Short-Form Health Survey) was used. We know that the SF-12, as well as the SF-36, are the most widely used QOL in studies on the ICD population internationally.^13^ However, SF-12 may not detect ICD specific QOL outcomes, especially mental health wellbeing, which proved to be a limiting aspect of this study.^11^

For example, the EFFORTLESS S-ICD Registry, in addition to the use of the SF-12 was careful to avoid possible bias attributable to the type of personality of the patient. For this, they used the DS 14. The authors took this care, as already indicated above, due to the knowledge that personality type D is a vulnerability factor in poorer QoL, life-threatening arrhythmias, and premature mortality in patients with an ICD.^14^

Another possibility would be for the author to use the SF-12v2 version, which also includes two summary measures, including a physical health component score (PCS) and a mental health component score (MCS).^11 , 15^

Finally, due to the important bias resulted from the re-operated patients after previous complications with a TV-ICD device, we consider that the comparative analysis of QoL between groups cannot be valued.

We expect that the continuous increase of S-ICD and prospective trials may be possible to reproduce these single-center observations and determine the best way to provide a safe, efficient, and comfortable anesthetic implant technique, always maintaining focus on the impacts on patients’ QoL.
